# A New Species of Charassothrips Hood from Colombia (Insecta, Thysanoptera, Thripidae) with an Updated Key to the Known Species

**DOI:** 10.1673/031.010.7001

**Published:** 2010-06-22

**Authors:** Arturo Goldarazena, Laurence Mound

**Affiliations:** ^1^ Neiker-Tecnalia, Basque Institute of Agricultural Research and Development, Department of Plant Production and Protection, Granja Modelo Arkaute, Antigua Carretera Nacional 1 km 255 E-01080 Vitoria-Gasteiz, Spain; ^2^ Honorary Research Fellow, CSIRO, Entomology, Canberra, ACT, 2601, Australia

**Keywords:** taxonomy, biodiversity, neotropical, *Charassothrips macroseta* new species

## Abstract

*Charassothrips macroseta* sp.n. is described and illustrated from Colombia. A key is provided to the five species now recognised in the Neotropical genus *Charassothrips*, each of which has the head and pronotum, mesonotum and metanotum prominently sculptured and the abdominal tergites with a craspedum on the posterior margins.

## Introduction

The Thysanoptera fauna of the South American country Colombia is poorly known. For example, the subfamily Thripinae includes 260 genera worldwide, but although 75 of these are recorded from Central America (Mound and Marullo 1996) only 20 genera have been reported from Colombia. This low number presumably reflects a lack of collecting activity, and systematic studies because the thrips fauna of Colombia is expected to be highly diverse, considering the topographical and floristic diversity of the country. Moreover, many taxa are known to be widespread between the countries of meso-America and Brazil, and this paper concerns one such genus.

The genus *Charassothips* was erected by Hood (1954) for a single species from Belem, Brazil, taken from the cylindrical inflorescence of an aquatic plant, *Urospatha caudata* (Araceae). Subsequently, the same species was found in Costa Rica breeding on cylindrical inflorescences of a related plant, *Urospatha friedrichstallii* (Mound and Marullo 1996). Johansen (1983) described a new genus *Humboldthrips* for two new species from Mexico, taken in the cylindrical inflorescences of species of *Piper* (Piperaceae). Both species were subsequently found in Costa Rica, co-existing in the inflorescences of a single species of *Piper*, and one of them was also found on a similar plant in southern Brazil (Mound and Marullo 1996). Moreover, because of the many similarities between the three thrips species, the genus *Humboldthrips* was synonymised with *Charasothrips* by Mound and Marullo (1996). Subsequently, Johansen (1996) described from Mexico a fourth related species taken from the forest canopy using an insecticide fogging technique.

The purpose of the present paper is to describe a further new species of *Charassothrips* from Colombia. This new species was taken from several different plants near Bogota, particularly from Asteraceae flowers that are very different in form from the known hosts of other species of *Charassothrips*. Unfortunately, there is no evidence that any of these was the host on which this thrips breeds.

## Material and methods

The specimens examined during this study were collected by L. A. Mound, and processed onto microscope slides at the Natural History Museum, London, using the standard procedure detailed on the web site (http://anic.ento.csiro.au/thrips/) and were then were identified and deposited in the British Museum of Natural History (BMNH). Samples of the three known species and the type series of the new species were borrowed and examined by A. Goldarazena. *Charassothrips leonilavazquezae* is known only from the male holotype and the information presented here is based on the original description. Dr. Johansen was unwilling to loan the holotype for security reasons. Information about the genus and other known species were obtained mainly from Mound and Marullo (1996). Measurements of the holotype and a male paratype of the new species were taken using a digital Leica 6500B microscope and the images were produced using differential interference contrast microscopy.

***Charassothrips* Hood**
*Charassothrips* Hood, 1954: 199. Type species *C. urospathae* Hood
*Humboldthrips* Johansen, 1983: 96. Type species *H. incomparabilis* Johansen, synonymised by Mound & Marullo, 1996: 106.
*Diagnosis*. Small brown or bicoloured macropterous species; antennae 7 or 8 segmented, segment 3 constricted at base (Figure 10), segments 3 and 4 each with forked sensoria. Head and thoracic nota with reticulate sculpture, reticles with or without short markings; setae short, lanceolate or acute. Pronotum with no long setae. Metanotal median setae arise behind or on anterior margin. Metanotum without campaniform sensilla. Tarsi 2-segmented. Mesothoracic spinula weakly developed; metathoracic furcal arms prolonged anterodorsally, no spinula. Forewing posteromarginal cilia wavy; setal row on first vein interrupted, with a long interval and 2 setae near wing apex; second vein with complete setal row. Abdominal tergal craspeda complete, lateral thirds either dentate or marginally smooth as central part (Figure 13). Sternites without discal setae. Male sternite III with glandular opening on anterior margin.
*Comments*. Johansen & Mojica-Guzman (1996) consider the glandular opening at the anterior margin of sternite II in males in this genus to be a “sucker-apparatus”, and conjecture that this “sucker adheres the male sternum to the female tergum...”. However, they present no behavioural evidence to support this, and the suggestion seems unlikely because thrips copulate side-by-side, attached only by the genitalia.

**Key to *Charassothrips* species**
1.- Antennae 7-segmented **2**
- Antennae 8-segmented **3**
2.- Forewing veinal setae broadly lanceolate, grooved longitudinally; metanotal median setae arising well behind anterior margin *C piperaffinis* Johansen
- Forewing veinal setae small; metanotal median setae arise on anterior margin aligned with lateral setae *C. leonilavazquezae* Johansen
3.- Abdominal segments uniformly brown; antennal segment II brown in contrast to yellow segment III; forewing setae slender and acute *C urospathae* Hood
- Abdominal segments III–VII white laterally; antennal segment II scarcely darker than III; forewing setae broad and grooved longitudinally **4**
4.- Female with pronotal sculptured reticles without internal markings (Figure 6); pronotum, mesonotum and metanotum with setae broad and grooved longitudinally; pronotum and abdominal tergites VIII–X mostly yellow *C. macroseta* n. sp.
- Female with pronotal reticles with internal markings; pronotum, mesonotum and metanotum with setae slender and acute; pronotum brown and abdominal tergites VIII–X brownish yellow *C. incomparabilis* Johansen

***Charassothrips incomparabilis* (Johansen)**
*Humboldthrips incomparabilis* Johansen, 1983: 104
*Charassothrips incomparabilis* (Johansen) Mound & Marullo, 1996: 108
The holotype and paratypes were collected on mosses and lichens growing on trunks of unknown trees in a mesophylous montane rain forest (Johansen 1983). This species was later captured more frequently in the inflorescences of *Piper auritum* and *P. aduncum* in Sierra Madre Oriental (Johansen 1996). *C. incomparabilis* has antennal segment V slightly constricted apically, whereas this segment is as broad as the base of VI in *C. urospathae*. The craspeda on tergites V–VI are dentate laterally, and the tergites have several lines of sculpture medially. The median setae on tergites II–IV are longer and closer together in specimens studied from Costa Rica than in those available from Mexico (Mound and Marullo 1996).
Specimens studied: 1 female and 1 male on *Piper* flowers, **Costa Rica**, La Selva, 27/04/1992 (LAM 2305).

***Charassothrips leonilavazquezae* (Johansen & Mojica-Guzman)**
*Humboldthrips leonilavazquezae* Johansen & Mojica-Guzman, 1996: 48
  This species is based to a single male collected using canopy fogging in the Tropical Deciduous Forest in Jalisco State. According to the description, the body is bicoloured with head, prothorax and abdominal segments II–III dark chesnut brown. In contrast, the pterothorax, middle and hind legs as well as abdominal segments I, IV and X are yellow. Males of *C. piperaffinis* and *C. incomparabilis* are similar to each other, and apparently differ from *leonilavazquezae*, in having the pterothorax brown, and abdominal segments III–VIII brown but sharply white laterally.

***Charassothrips piperaffinis* (Johansen)**
*Humboldthrips piperaffinis* Johansen, 1986: 724
*Charassothrips piperaffinis* (Johansen) Mound & Marullo, 1996: 108
*C. piperaffinis* is very similar in colour to *C. incomparabilis*. This species was collected from the same *Piper* species as *C. incomparabilis*, but the long ovipositor of females might indicate that eggs are laid in a different position on the flowers in these two species (Mound and Marullo 1996).
Specimens studied: 2 females and 2 males on *Piper* flowers, **Costa Rica**, La Selva, 25/11/1992 (LAM 2421).

**Figures 1–7.**
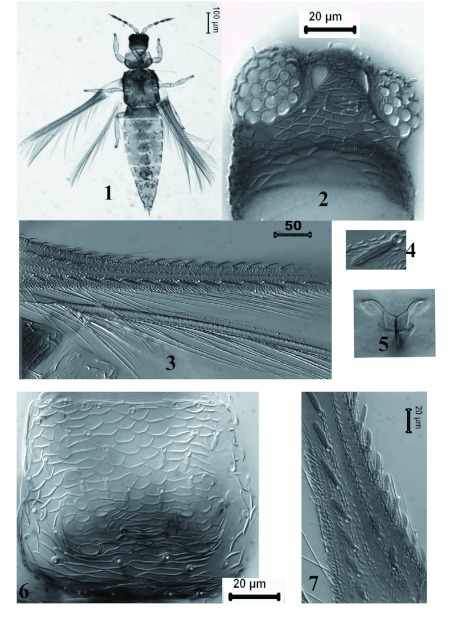
*Charassothrips macroseta female*. 1, Habitus. 2, Head. 3 and 7, Forewing.4, Forewing first vein setae. 5, Metathoracic furca. 6, Pronotum. High quality figures are available online.

**Figures 8–13.**
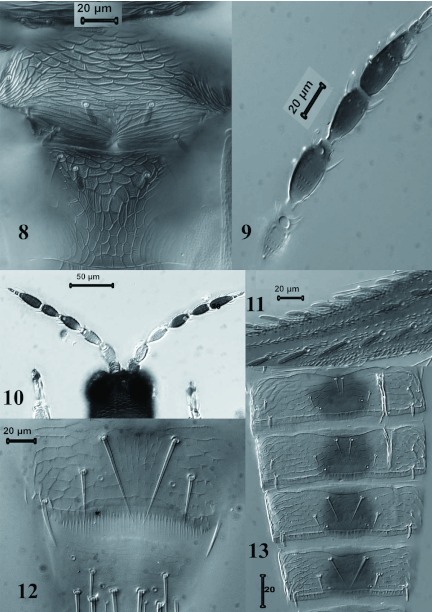
*Charassothrips macroseta* female. 8, Mesonotum and Metanotum. 9–10, antenna. 11, Forewing costal setae. 12, Abdominal tergite VIII. 13, Abdominal Tergites III–VI. High quality figures are available online.

**Figures 14–19.**
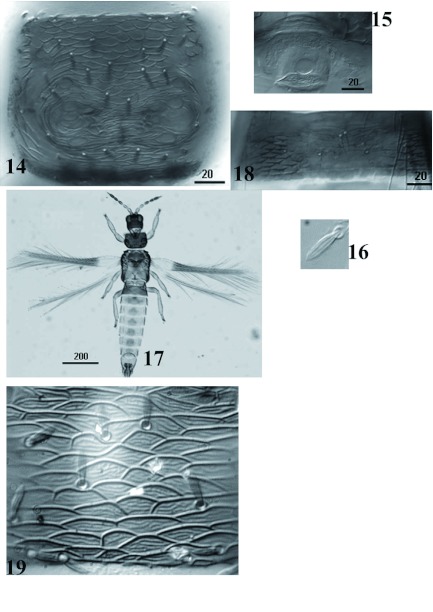
*Charassothrips macroseta* male. 14, Pronotum. 15, Glandular pore. 16, Forewing seta broad and grooved longitudinally. 17, Habitus. 18, Tergite III with craspedum. 19, Pronotal disc with polygonal sculpture well developed, with internal markings. High quality figures are available online.

***Charassothrips macroseta* sp. nov. (Figures 1–19)**
Female macroptera (Fig 1–13)
Body bicoloured (Figure 1); head, mesonotum, metanotum, abdominal segments I–II and medial area of abdominal segments III–VII brown; pronotum, lateral areas of abdominal tergites III–VII, tergites VIII–X and legs yellow; antennal segment I yellowish brown, II–III yellow, IV–VIII brown with bases of IV–V pale (Figures 9–10); forewing sharply pale at base in contrast to brown distal three quarters.
Head wider than long (Figure 2), cheeks slightly incut behind eyes, dorsal surface with heavy sculpture with polygonal reticulation; ocelli well developed with three pairs of minute ocellar setae; ocellar setae III on margins of triangle. Antennal segment I small and quadrangular, II with pair of grooved dorsal setae lateral to campaniform sensillum and one setae nearer base; III and IV with sensorium forked; V and VI each with two simple sensoria laterally; VII and VIII clearly differentiated.
Pronotum rectangular without long setae (Figure 6), discal setae broad and grooved longitudinally; pronotal disc with polygonal sculpture well developed, without internal markings. Mesonotum with polygonal reticulation and 3 pairs of broad setae (Fig 8). Metanotum with polygonal reticulation; anterolateral setae slender and acute, median pair broad and arising well behind anterior margin (Figure 8); campaniform sensilla absent. Metapre-episternum well-developed. Metathoracic furca with two arms prolonged anterodorsally (Figure 5). Forewing clavus with 7 broad veinal setae and one broad discal seta; first vein with 8 basal and 2 distal setae, second vein setal row complete with 20 setae; all veinal setae grooved longitudinally (Figures 3, 4 and 7).
Abdominal tergites II–IV with median setal pair long and close together; tergites II–VII with 3 broad setae on lateral areas; lateral thirds of tergites with small dentate microtrichia on sculpture lines (Figure 13); craspeda well developed, dentate laterally on tergites V–VI (Figure 13); tergite VIII with comb of microtrichia complete (Figure 12). Sternites without discal setae. Sternite II with four marginal setae, III–VI with 6 marginal setae, and VII with 2 marginal and 2 discal setae.
Measurements in micrometers (Holotype female). Body length 1487. Head; dorsal length 80; width 110. Ocellar setae 3. Pronotum length: 123, median width 174. Pronotal major setae: 9–10. Discal setae: 11. Mesonotal setae: 13–13.5. Anterior lateral setae of the metanotum: 22. Metanotal median setae: 13.6. Forewing length: 796. Forewing costal setae: 15–20. Second vein setae: 22–29. Pair of median setae of the abdominal tergites II–IV: 27–28. Tergite IX setae B1 58.5, B2 79.4, Tergite X length 63.5. Marginal setae of sternites II–VII: 28–32. Antennal segments I–VIII length: 19; 32; 47; 44; 36; 43; 8; 12.
Male macroptera (Figures 14–19). Similar in color to female. Pronotal disc with polygonal sculpture well developed, with internal markings (Figure 19) and setae broad and grooved longitudinally (Figure 16). Smaller than female (Figure 17); sternite III anterior margin with glandular pore about 18 microns diameter (Figure 15). Measurements: Body length 1269. Head length 72.7, width 100. Pronotum (Figure 14), length 109, width 155. Anteroangular, anteromarginal, posteroangular and posteromarginal setae minute 7–9, discal setae 13. Forewing length 197 (Figure 17). Antennal segments I–VIII length: 13; 30; 42; 40.5; 30; 42; 7; 11.

## Material studied

Holotype female, **Colombia**, Cota 2700 m altitude, near Bogotá, from *Baccharis* flowers (Asteraceae), 10.vii.1993 (LAM 2465) in (BMNH).

Paratypes: 11 females 6 males with the same data as the holotype — 9 females, 6 males in (BMNH) 1 female in National Museum of Natural History, Washington D.C, and 1 female in Australian National Insect Collection, CSIRO Entomology, Canberra, Australia. **Colombia**, Cota 2700 m altitude near Bogotá, 1 female, 1 male from *Cytisus* flowers, 10.vii.1993 (LAM2463); same locality, 1 female on *Phyllanthus* flowers, 10.vii.l993 (LAM2464).

***Charassothrips urospathae* Hood**
*Charassothrips urospathae* Hood, 1954: 200
This species was collected originally at Belem, Brazil on *Urospatha caudata*. The abdominal craspeda bear marginal microtrichia laterally, but the teeth of the comb on tergite VIII are unusual in being long and slender but with a relatively broad, parallel-sided base. The head, prothorax, pterothorax and abdominal segments are brown.
Specimens studied: 2 females and 2 males on the spadix of *Urospatha friedrichsthallii*, **Costa Rica,** La Selva, 27/11/1992 (LAM 2426).

## Discussion

Variation between species within this genus is particularly interesting, involving the number of antennal segments, the form of the major setae, the body colour, and the markings within the surface reticulation. *C. macroseta*, *C. incomparabilis* and *C. urospathae* each have eight antennal segments, whereas *C. leonilavazquezae* and *C. piperaffinis* have seven segments. *C. urospathae* has the abdominal segments uniformly brown, but *C. incomparabilis* and *C. macroseta* have abdominal segments III–VII white laterally with a brown spot medially. In *C. incomparabilis*, the brown spot is irregular, semicircular and bigger than in *C. macroseta*, where it is circular. This character might be dependent on the developmental stage of the thrips, but all the examined specimens of *C. macrosetae* have the same circular spot in the central area of the abdominal segments. Abdominal segment VIII is brown in *C. incomparabilis* but yellow in *C. macroseta*. The pronotal sculpture also differs between and within species. Females of *C. incomparabilis* have markings within the pronotal reticles whereas these are not present in *C. macroseta*. Males of *C. incomparabilis* and *C. macroseta* share that character. The pronotal, mesonotal and metanotal setae are bigger and clearly grooved longitudinally in *C. macroseta*, whereas in *C. incomparabilis* these setae are slender and acute.
